# Contrasting Nephropathic Responses to Oral Administration of Extract of Cultured *Penicillium polonicum* in Rat and Primate

**DOI:** 10.3390/toxins2082083

**Published:** 2010-08-09

**Authors:** Peter G. Mantle, Katharine M. McHugh, John E. Fincham

**Affiliations:** 1 Biochemistry Department, Imperial College, London SW7 2AZ, UK; Email: katy.mchugh@tiscali.co.uk; 2 Research Institute for Nutritional Diseases, Medical Research Council, Tygerberg 7505, South Africa; Email: john@uninet.co.za

**Keywords:** karyomegaly, karyocytomegaly, porcine nephropathy, *Penicillium polonicum*, ochratoxin A, mycotoxin, vervet monkey, rat renal pathology

## Abstract

Liquid- or solid substrate-cultured *Penicillium polonicum* administered in feed to rats over several days evokes a histopathological response in kidney involving apoptosis and abnormal mitosis in proximal tubules. The amphoteric toxin is yet only partly characterized, but can be isolated from cultured sporulating biomass in a fraction that is soluble in water and ethanol, and exchangeable on either anion- or cation-exchange resins. After several weeks of treatment renal proximal tubule distortion became striking on account of karyocytomegaly, but even treatment for nearly two years remained asymptomatic. Extract from a batch of solid substrate fermentation of *P. polonicum* on shredded wheat was incorporated into feed for rats during four consecutive days, and also given as an aqueous solution by oral gavage to a vervet monkey daily for 10 days. Treatment was asymptomatic for both types of animal. Rat response was evident as the typical renal apoptosis and karyomegaly. In contrast there was no such response in the primate; and neither creatinine clearance nor any haematological characteristic or serum component concentration deviated from a control or from historical data for this primate. The contrast is discussed concerning other negative findings for *P. polonicum* in pigs and hamsters. Renal karyomegaly, as a common rat response to persistent exposure to ochratoxin A, is not known in humans suspected as being exposed to more than the usual trace amounts of dietary ochratoxin A. Therefore the present findings question assumptions that human response to ochratoxin A conforms to that in the rat.

## 1. Introduction

*Penicillium polonicum* K. M. Zalessky includes a group of fungi previously included within the broad species *P. aurantiogriseum* Dierckx, and historically within *P. verrucosum* var. *cyclopium*. Revision in the 1990s differentiated several distinct groups, for which refined cultural protocols for consistent expression of particular phenotypic characters, combined with some closely associated idiolytes, has created more meaningful speciation [1,2,3,4]. We have direct experience of occurrence of verrucosidin and penicillic acid [5], auranthine [6], anacine [7]. Other metabolites of an isolate of *P. aurantiogriseum* from the same Yugoslavian village as that used here are quoted as penicillic acid, fructigenine A, cyclopenol, cyclopenin, cyclopeptin, dehydrocyclopeptin, viridicatol, 3-methoxy-viridicatin, rugulosuvine, verrucofortine and normethylverrucosidin [7].

Concurrently, toxicological interest in some isolates of *P. verrucosum* var. *cyclopium* arose from the seminal findings in experimental rats of a nephropathy involving marked karyomegaly in the *pars recta* region several days after administration of cultured mycelium [8]. The findings were offered as of potential etiological relevance to the idiopathic human disease Balkan endemic nephropathy, since the fungus can readily be found as a stored-food spoilage mould in the region.

Further study has shown the rat nephropathy caused by moulds historically described as *P. verrucosum* var. *cyclopium* or *P. aurantiogriseum* or *P. commune* [9,10] to be confined to those now defined by the recent restoration of *P. polonicum* [4]. Chemical characterisation of the nephrotoxin is still incomplete, although it is water-soluble and amphoteric, and may be a peptide [11]. Weanling rats of both sexes are susceptible, responding to ingestion of contaminated feed within a few days by mitoses in proximal tubule epithelium progressing to striking diffuse karyocytomegaly distributed mainly throughout the outer stripe of the outer medulla [12,13,14,15]. In contrast, there was no similar response in the hamster [16]. A renal apoptotic component in early responses to oral administration to rats of cultured *P. polonicum*, or an extract, has been demonstrated [17].

Opportunity to make direct comparison of toxicological response to several days of treatment with a *P. polonicum* culture extract in rat and vervet monkey arose 20 years ago. Significance of the result was difficult to perceive at the time. However, there is recent suggestion of involvement of the fungus in porcine nephropathy in Bulgaria and South Africa [18,19]. 

## 2. Materials and Methods

### 2.1. Preparation of fungal extract for toxicity test

Shredded wheat breakfast cereal biscuits were crushed to the extent that 60 g was easily transferred via a paper funnel to each of sixty 500 mL conical flasks. 30 mL distilled water was added to each flask, cotton wool plugs added, flask contents homogenised by shaking and then steam sterilised at 120 °C for 20 min. Spores of isolate M6 [10] were added. Flasks were shaken to homogenise and incubated at 18 °C for 2 weeks. Flasks were shaken again on days 3 and 8 to homogenise and aerate the moulding mass, which gradually became blue/grey with spores. On completion, a single flask was designated for a separate test with rat 1 (below). Ethyl alcohol (95%, 200 mL) was added to each of the other 59 flasks, to wet the spores and disrupt cellular membrane integrity, shaken and left overnight to extract. Supernatants were decanted and combined (~5 L) and a similar volume of 95% ethyl alcohol was replaced throughout the flasks. After soaking overnight, extracting mixtures were transferred to large Buchner filters and the liquid phase drawn-off *in vacuo* (12 L). Residues were treated with distilled water (8 L) and aqueous extract removed *in vacuo*. Alcoholic and aqueous extracts were combined and stirred with cation-exchange resin generated in the H^+^ form. Duolite cation-exchange resin (500 g, 100 mesh) was generated in the H^+^ form by batch treatment with 2 N HCl. After washing with distilled water until in an environment of pH > 6 was achieved, the resin was added to combined culture extract in batches (5 × 100 g) until extract ceased to become acidic in response to H^+^ exchange. Thereby cation-exchangeable components were separated from other components of the shredded wheat fermentation (e.g., fungal sterols, unused wheat saccharides and acidic substances. Resin was separated, washed and treated with 2 N NH_4_OH to release bound cation-exchangeables. The eluate was evaporated *in vacuo* to a small volume and then freeze-dried (51.25 g). The product therefore consisted of compounds that were soluble in both water and alcohol and that could bear a positive charge. This represented an amount equivalent to ~1.5% of the weight of the original substrate after exhaustive biotransformations by *P. polonicum* and would have excluded penicillic acid, verrucosidin, auranthine, anacine and neutral polar compounds such as sterols. 

### 2.2. Rat experimental

Male Sprague-Dawley rats (~250 g) were given 20 g powdered diet (Rat and Mouse No. 1; Special Diet Services, Witham, Essex, UK) into which was homogenised either a 20% component of shredded wheat moulded by *P. polonicum* (Table 1, rats 1, 4 and 5) or an aqueous solution of the extract described above (rats 2 and 3). Experimental conditions were as previously described [12]. Unilateral (left) nephrectomy was done on rat 5 after 10 weeks of treatment with the *P. polonicum* diet so that histological evidence could be made at this stage. After two weeks post-operative recuperation the rat was returned to the *P. polonicum* diet. All experimental animal procedures were in accord with a licence issued by the UK Home Office. 

**Table 1:** Summary of rat treatments with whole cultured *Penicillium polonicum* or extract.

**Rat**	**Period of exposure**	***P. polonicum* treatment format**	**Daily consumption of nephrotoxin, expressed as weight of shredded wheat substrate moulded, and consumed, whole or as extract in feed**
1	4 days	Moulded shredded wheat	5 g
2	4 days	Water-soluble extract fraction	7 g
3	4 days	Water-soluble extract fraction	35 g
4	5 weeks	Moulded shredded wheat	5 g
5	10 weeks, and more	Moulded shredded wheat	5 g

### 2.3. Primate experimental

Faeces and urine were examined daily macroscopically and microscopically, respectively. “Combur^9 ^Test” strips (Boehringer, Mannheim, Germany) were used daily for fresh urine; specific gravity was measured with a refractometer. Haematology and urology analysis was by standard methodology in the primate unit. 

Two young male vervet monkeys (*Chlorocebus aethiops*), 19 and 21 months old (2.4 and 2.33 kg, respectively) were selected as treated and control subjects. Their food was prepared in-house based on corn porridge with added protein, vitamins and minerals, and with a daily portion of fruit (either citrus or apple). Food and water was available *ad libitum*. The treated animal was given the fungal culture extract (100 mL) daily by nasogastric intubation without sedation, subdivided into 10 equal doses on consecutive days. The control received distilled water (10 mL) similarly, daily and concurrently. There was no regurgitation after dosing. Before and after the dosing regimen both animals were sedated with ketamine (10 mg/kg body mass) for measurement of body weight, rectal temperature, respiration rate and pulse rate. 

Surgical anaesthesia was induced with intramuscular ketamine, 10 mg/kg (Warner Lambert) and atropine, 0.05 mg/kg, and maintained by inhalation of a mixture of fluothane (ICI, Halothane)-nitrous oxide-oxygen (2:40:58, v/v). Inhalation was through an endotracheal tube connected to a semi-closed circuit which included a ventilator. Respiration was supported by ventilation from before the thorax was opened, until after death. Isotonic saline containing 500 units of heparin per 200 mL was dripped into a saphenous vein, starting when anaesthesia was induced. The saline was continuously oxygenated.

The thorax was opened by saggital mid-sternal incision and infusion of oxygenated heparinised saline was commenced into the left ventricle through a 14 gauge needle at a pressure of 100 mm Hg. The capsule was stripped from kidneys and the cortex was incised along the saggital axis into the medulla. Transverse cuts were made through the cortex at approximately 3 mm intervals. The saline was also infused into the left common carotid artery through a 21 gauge catheter. The right artery was tied off, and the jugular veins were cut. The vasculature was flushed until haemodilution was evident in the outflow from the incised kidneys and jugular veins. This procedure prevented formation of intravascular agonal clots.

When haemodilution was achieved, the saline was stopped and perfusion into the heart and brain of oxygenated fixative consisting of 1% (v/v) glutaraldehyde and 4% formaldehyde commenced. Perfusion into the arterial system continued for 15 min and circulated between 500 and 1000 mL of fixative. The heart continued beating for a few minutes after perfusion of fixative commenced and ventilation was maintained during this time. After perfusion, the kidneys were excised and immersed in neutral buffered formalin (10%, pH 7.2). Representative cross-sections of each kidney were embedded in wax and standard histology sections were prepared, stained with haematoxylin and eosin. The study was approved by the Animal Ethics Committee of the South African Medical Research Council.

## 3. Results

### 3.1. Rat: Demonstration of renal histopathogenic equivalence of whole fungal culture and the extracted fraction after short-term exposure

A nephrotoxicity test on one randomly-selected flask culture from the batch of 60, giving one-third of the flask contents (representing 20 g of moulded shredded wheat) in feed to rat 1 over 4 days, caused marked renal karyomegaly similar to that previously described [8,11,15]. 

**Figure 1:**
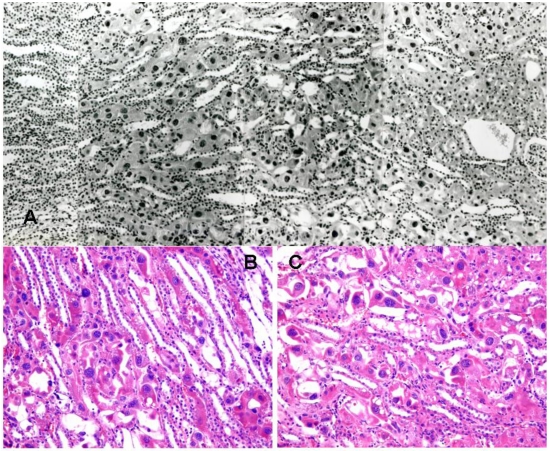
Rat renal histopathology (stained with H & E) after five weeks on a *P. polonicum*-contaminated diet (rat 4). A, part of saggital section traversing from innermost glomerulus in cortex (right) into the medulla, showing extensive, diffuse karyocytomegaly across the region. B and C, two representative examples in the region associated with concentration of P3 segments of nephrons, showing detail of enlarged nuclei in distorted cells.

A similar degree of diffuse histopathological change was produced in rat 2 by giving similarly in feed a small sample of the extract from the remaining 59 flasks, most of which was used later for the primate. The total amount of extract given to rat 2 over the four days represented 28 g of shredded wheat culture, implying ~95% efficiency in retaining the nephrotoxin(s) through selection of a fraction of alcohol-soluble and cation-exchangeable components of the fermented cereal substrate. Increasing the dose of extract five-fold for rat 3 only gave slightly greater incidence of the characteristic histopathological change, indicating that the lower dose was already near maximum for effect.

### 3.2. Rat: Renal histopathology after five, ten or sixty-six weeks on a diet containing a 20% component of shredded wheat moulded by *P. polonicum*

**Figure 2:**
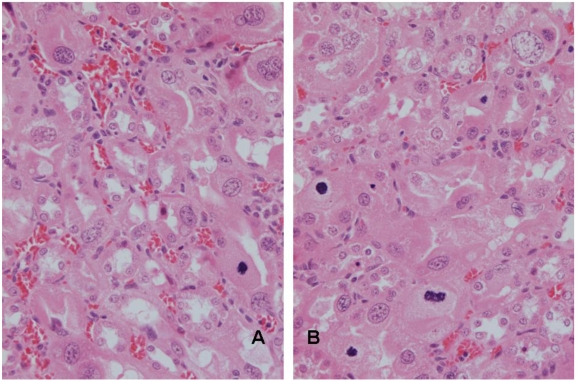
A,B. Renal histopathology of rat 5 after 10 weeks exposure to *P. polonicum*-contaminated diet, showing karyomegalic nuclei with prominent nucleoli in extensively distorted epithelium of P_3_ segments of nephrons. Mitoses, indicated by the condensed chromatin, reflect current mitogenic influence of the mycotoxin. Some cells have several nuclei.

**Figure 3:**
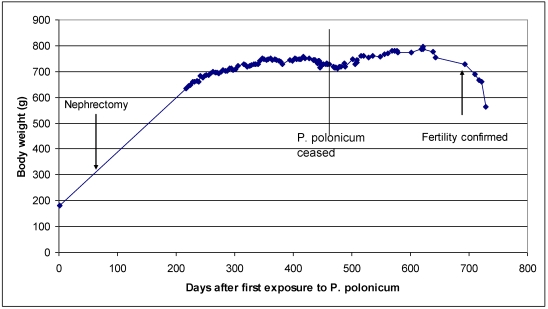
Body weight of rat 5 during and after exposure to *P. polonicum*, indicating stages of unilateral nephrectomy and confirmation of fertility.

In rat 4, after five weeks on the *P. polonicum-*contaminated diet, the karyomegaly seen clearly in rats 1–3 above after four days had progressed in magnitude and frequency to the striking karyocytomegaly illustrated in Figure 1. After ten weeks treatment for rat 5, unilateral nephrectomy provided tissue for histology showing further progression of karyocytomegaly while mitotic events continued (Figure 2 A, B). Monitoring body weight in rat 5 (Figure 3) showed that *P. polonicum* was well tolerated for a total of 463 days since first exposure. Permanent transfer then to normal feed coincided with foot lesions caused by maintaining a heavy animal on paper, reflected in the temporary decline in body weight (Figure 3) while lesions healed. Notably, fertility was confirmed 693 days after *P. polonicum* exposure commenced (25 months of age); five weeks later the rat was found moribund and euthanized. The only notable feature at necropsy was the remaining kidney (9.4 g, 3.3 cm long axis), much enlarged at least to compensate for the previous unilateral nephrectomy. The kidney surface implied extensive cysts, which are commonly a feature of ageing. Histology revealed the cysts, and persistent karyocytomegaly in the cortico-medullary region, but there was no evidence of tumour. 

These tests served to verify the nature of the renal histopathological target and the potency of the experimental culture of *P. polonicum* in the rat, for the purpose of comparing the effect of oral administration to the vervet monkey.

### 3.3. Vervet monkey

Stools of the treated animal darkened two days after treatment commenced; this is not surprising since the extract was brown coloured. Body weight, temperature, respiration and pulse in treated and control animal were stable over the 10 day treatment period (Table 2).

**Table 2:** Body weight of vervet monkeys, rectal temperature and rates of respiration and pulse before and after treatment with *P. polonicum* extract.

**Clinical**	**Before treatment**	**After treatment**
Body wt (kg)		
Treated	2.40	2.40
Control	2.33	2.24
Rectal temp (°C)		
Treated	39.0	39.0
Control	38.4	38.8
Respiration (/min)		
Treated	42	40
Control	36	28
Pulse (/min)		
Treated	180	180
Control	180	160

Microscopicallv, fresh urine contained no red blood cells, white blood cells, casts, crystals or bacteria. Epithelial cells, probably from bladder, were rare. Mean urine pH value throughout the treatment period was 6.0 ± 1.1 and 7.2 ± 1.4 for treated and controls, respectively. Similarly, specific gravity was 1.015 ± 0.011 and 1.014 ± 0.009, respectively. Tests for leukocytes, nitrite, protein, glucose, ketones, urobilinogen, bilirubin and blood showed no significant difference between the animals. Creatinine clearance, as a sensitive indicator of glomerular and tubular function, was consistently within the normal range (Table 3).

**Table 3:** Creatinine clearance as an indicator of glomerular and tubular function (mL of serum cleared of creatinine per kg body weight per minute).

	**Three weeks before treatment**	**Penultimate treatment day**	**Last treatment day**	**Reference data** (n = 74 *)
**Treated**	2.72	2.67	2.99	2.43 ± 0.56
**Control**	3.09	2.09	2.69	2.43 ± 0.56

* For healthy, age and sex-matched vervets in the Research Institute for Nutritional Diseases primate unit.

Concerning haematology, mild reduction in haemoglobin occurred in both cases but, generally, nothing in the haematology findings supports any toxic effect (Table 4). Concerning serum chemical pathology (Table 5), the variable which closely reflects renal function is creatinine, which remained constant for the treated animal. Changes in electrolytes or urea must occur with elevation in creatinine concentration to link them to renal malfunction, because alone they are non-specific. The moderate increases in aspartate transamidase (AST), alanine transamidase (ALT), lactate dehydrogenase (LD) and alkaline phosphatase (ALP) in both animals are attributed to muscle strain generated during the daily physical restraint for dosing and the accompanying reflex adrenergic discharge. 

**Table 4:** Comparative haematology data, before and near the end for treated (T) and control (C).

	RBC	Hb	Hct	MCV	RDW	Plt	WBC	N	L
**T 4 weeks before treatment**	5.25	13.0	38.6	73.5	24.0	467	7.05	2.23	4.50
**T penultimate treatment day**	5.09	12.6	37.6	73.8	20.0	425	5.26	1.96	3.00
**T last treatment day**	5.02	11.7	37.4	74.5	23.3	429	6.90	2.66	3.89
**C 4 weeks before treatment**	5.21	13.2	40.0	76.7	23.0	338	3.26	1.07	1.92
**C penultimate treatment day**	5.00	12.8	38.3	76.5	18.8	271	7.52	6.01	0.97
**C last treatment day**	5.15	12.3	39.5	76.7	19.8	297	3.65	1.80	1.51

RBC = red blood cells × 10^6^/mL; Hb = haemoglobin g/dl; Hct = haematocrit (packed red cell volume)%; MCV = mean corpuscular volume (red cell volume) µ^3^; RDW = diameter variation in red cells%; Plt = platelets × 10^3^/mL; WBC = white blood cells × 10^3^/mL; N = neutrophils × 10^3^/mL; L = lymphocytes × 10^3^/mL.

No significant histopathological change was found in kidneys, in which tubular conformation had been conserved by the perfused fixation. It is concluded that in a young male vervet the *P. polonicum* extract was not detectably nephrotoxic by the applied methods at the dose used within 10 days.

**Table 5:** Comparative serum chemical pathology data for treated (and control) vervets before (T1, C1) and near the end of (T2, T3; C2, C3) treatment with *P. polonicum* extract. Sample times as in Table 4.

	**T1**	**T2**	**T3**	**C1**	**C2**	**C3**	Reference *
**Sodium mmol/L**	149	154	146	148	156	149	149.9 (2.8)
**Potassium mmol/L**	3.8	3.7	3.5	3.9	3.6	3.8	3.5 (0.6)
**Chloride mmol/L**	112	115	108	100	110	105	108.5 (2.2)
**CO_2_ mmol/L**	27	27	26	26	23	31	25.7 (4.0)
**Anion gap mmol/L**	10	12	12	13	23	13	15.4 (4.8)
**Urea mmol/L**	4.6	8.9	12.5	3.9	17.1	10	5.4 (1.7)
**Creatinine µmol/L**	71	71	71	71	124	80	67.7 (17.9)
**Total protein g/L**	62	63	62	60	61	58	61.3 (4.6)
**Albumin g/L**	42	43	43	42	42	41	39.6 (3.6)
**Globulin g/L**	20	20	19	18	19	17	21.7 (5.1)
**Calcium mmol/L**	2.33	2.33	2.30	2.38	2.33	2.30	2.23 (0.2)
**Phosphorus mmol/L**	1.84	1.74	1.68	1.97	2.00	1.55	1.97 (0.4)
**Magnesium mmol/L**	0.60	0.69	0.70	0.61	0.88	0.66	-
**Cholesterol mmol/L**	3.77	3.82	4.06	5.15	5.72	5.75	4.4 (1.9)
**Bilirubin µmol/L**	3	3	3	3	7	5	3.2 (1.8)
**Unconjugated Bilirubin µmol/L**	3	3	3	3	7	5	2.7 (1.0)
**Glucose mmol/L**	6.5	4.5	4.7	5.4	4.4	4.5	4.1 (1.1)
**AST units/L**	47	59	96	36	24	72	92.8 (69.8)
**ALT units/L**	66	97	101	89	90	57	65.6 (53.0)
**LD units/L**	350	371	547	348	294	708	-
**GGT units/L**	52	48	45	63	68	63	61.6 (47.3)
**ALP units/L**	880	894	1007	1050	1314	1303	824 (515.1)

* Means and (standard deviations) for young males in the Research Institute for Nutritional Diseases primate unit (n = 62).

## 4. Discussion

The total cumulative dose given to the vervet over the 10 day period exceeded that given to a rat by a factor of 5.85 on a body weight basis, and by a factor of 2.3 expressed also on a daily basis. A further two-fold factor might even be applicable to widen the difference on a metabolic rate basis. Thus, the negative response in the primate is the more compelling.

Studies were made over 20 years ago on putative toxicity of *P. polonicum* to pigs because of its abundance as a food spoilage mould [11,20], and because the toxin causing renal responses in the rat resides in spores [10]. However, until now, the consistent negative findings were not easy to report. Relatively large amounts of cultured *P. polonicum* were used in collaboration in the UK with Prof. R.H.C. Penny at the Royal Veterinary College, London, UK and Dr. J. Heaton, Consultant Pathologist, Victoria Hospital, Worksop, UK.

In 1988, 750 g (wet weight) cultured mycelium of an isolate from a Yugoslavian village in which Balkan nephropathy was hyperendemic [9] was given to each of two 16 kg pigs, subdivided over five daily feeds (approx 30 g dry weight of fungus), in a slurry disguised with milk powder. No renal histopathological changes occurred. Similarly, daily dose of ~40 g (dry weight) of Yugoslavian isolate M2 [9] also evoked no renal histopathology. In 1989, administration over six days of isolate M2 to two adult mini-pigs (10 kg) also failed to reveal renal toxicity. In 1990, extract of yet another Yugoslavian isolate M6 [10], as also used here, was given in feed to another 15 kg pig over seven days; no renal histopathology was found. This contrasts with demonstration that only 1 g of *P. polonicum* spores caused typical acute rat renal histopathological change when administered in feed over four days.

Renewed published interest in *P. polonicum* stems from common occurrence of this mould in feed for pigs, not only in Bulgaria [19], but also in S Africa [18], where similar cases of idiopathic porcine nephropathy have been recognised. Isolation and partial characterisation of a highly non-polar metabolite of *P. polonicum* and other fungi [21] has led to speculation that this compound may be the *P. polonicum* metabolite which caused the striking rat renal karyocytomegaly illustrated here, and could be a factor in a putative multi-mycotoxin etiology for the porcine nephropathy. Unfortunately no elemental composition was proposed for the molecular mass of the metabolite (390.27701). The closest elemental composition is C_24_H_38_O_4_, which fits several sterols; that type of compound could also fit the non-polar chromatographic mobility. Toxicity towards human lymphocyte cells was shown [21], but unfortunately without any control. Nevertheless, omission to cite [12] could account for not appreciating that the rat renal response to whole *P. polonicum*, or an extract designed to be cation-exchangeable and thus to exclude sterols and acids, could hardly be due to that highly non-polar compound. 

It so happens that another mycotoxin that causes karyomegaly in rat renal proximal tubules is ochratoxin A, which was first recognised as a mycotoxin in South Africa [24]. Ochratoxin A is much less active in causing karyomegaly in male rats, requiring longer exposure and never achieving the magnitude of the *P. polonicum* toxin [12](Figure 3). Aneuploidy was recognised for both ochratoxin A and *P. polonicum* toxin and measured for the latter as incomplete multiples of the genome even beyond octaploid [25,26].

In a study in which pig feed contaminated with ochratoxin A at 800 µg/kg was given continuously for a whole year [22], only slight kidney hypertrophy ensued, which would probably have been unrecognisable in routine slaughterhouse processing. Thus the ~70 µg/kg measured in commercial feed for bacon pigs (for 6–8 months only) in two years in South Africa [18], hardly expected to be significantly nephrotoxic, would seem to require a very exceptional synergism with other natural toxicants to cause significant porcine nephropathy there. Consequently, the evidence [18] could equally be interpreted as that nephropathy not being a mycotoxicosis. The porcine nephropathy also seen in Bulgaria, associated with feed contaminated variously at ~100–200 µg ochratoxin A/kg in three years, and at 375 µg/kg in another [19,23], would still require considerable augmentation by other mycotoxins, and any contribution from *P. polonicum* would appear to be implausible. Nevertheless the matter could readily be resolved by experiment.

There is no suggestion that a karyomegalic nucleus is already *en route* towards renal tumourigenesis. However, demonstration of the aneuploidy in rat kidney in response to five weeks of continuous maintenance on feed contaminated with an ~20% component of shredded wheat moulded by *P. polonicum* has defined the extent to which the ‘polyploid’ nuclei illustrated in Figure 1 can be composed of a high proportion of incomplete genome multiples. Since the karyocytomegaly illustrated in Figure 2 persists for life [12], and aneuploid nuclei are generally recognised as having tumourigenic potential [26], the abnormal organelles may harbour genetic damage, including that resulting from mis-repaired adducts between DNA and ochratoxin A [27] or from any other putative mechanism proposed [28]. In the case of ochratoxin A, it is reasonable to consider that certain tumourigenic permutations of randomly-generated genetic change could reside in some of the karyomegalic nuclei, which are located in the rat kidney region within which renal tumours seem to arise.

**Figure 4:**
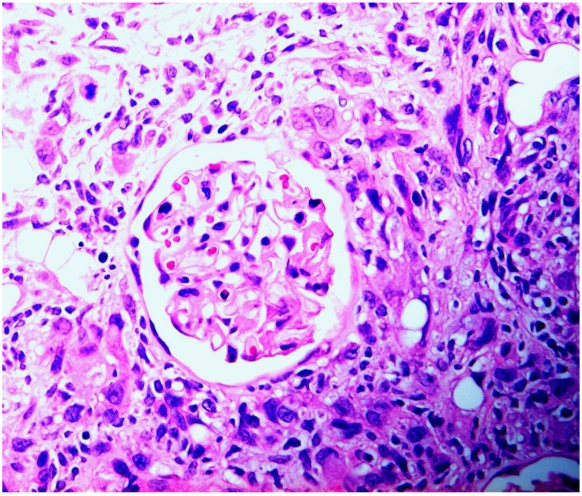
Rat renal carcinoma, characterized by disorganized proliferation of cells with pleomorphic nuclei, infiltrating (left) into cortex and enveloping an intact glomerulus.

The present situation with respect to nephrotoxicity of *P. polonicum* has echoes of a mycotoxicosis caused by *Fusarium verticilliodes*, described in its first report [29] as hepatocarcinoma and oesophageal basal cell hyperplasia in rats fed an artificially moulded dietary component. The finding generated wide interest, particularly in the USA, because the fungus readily causes spoilage of maize. There was also a regional hypothesis concerning putative involvement in hotspots of human oesophageal cancer in the Republic of Transkei. Subsequent studies in South Africa recognised the new mycotoxin fumonisin B1 as causing diverse toxicological responses in horse (leukoencephalomalacia), pig (pulmonary oedema) and rat (renal carcinoma). For the latter, a comprehensive 2-year toxicology study has been made [30], revealing male-specific renal carcinoma. Concurrently, Hard *et al.* [31] stated “carcinomas induced by fumonisin B1 were predominantly a rare and highly malignant variant of renal tubule tumour capable of infiltrative growth invading between tubules and glomeruli, sequestering these pre-existent elements as entrapped remnants within the tumour mass.” Much the same could be said concerning male rat renal tumours in response to ochratoxin A, with respect to envelopment of glomeruli (Figure 4). Hard *et al.* [31] also recognised sarcomatoid areas in the National Toxicology Program’s fumonisin renal tumours; a similar sarcomatoid component of mammary tumour has also recently been recognised in a female rat given the tumour promoter sodium barbitate in drinking water after chronic dietary exposure to ochratoxin A [32]. 

However, when a toxicology study was made during 13 years with the same fumonisin-producing *Fusarium* as that described by Marasas and colleagues in a non-human primate, no adverse histopathological renal or oesophageal changes were found [33], and current research does not easily sustain a causal hypothesis for fumonisin in oesophageal cancer. The present contrasting findings with *P. polonicum*, also in the vervet monkey, extend uncertainty about the rat as a good model for the human with respect to ochratoxin A as a potential renal carcinogen. This is compounded by the negative responses also in the pig.

Unfortunately, ochratoxin A seems never to have been tested for any renal histopathological changes in the primate. Toxicokinetics in the vervet monkey was studied [34] for up to 21 days after a single dose (2 mg ochratoxin A/kg), revealing a plasma half-life of that duration. However, although slow release from plasma proteins to renal elimination was occurring over that period, opportunity to study renal histopathology was unfortunately omitted. The failure also of hamsters to show any renal response, in an elegant study [16] of perfuse-fixed kidneys from animals given the same fungus as used here for up to five weeks, contrasts with the present striking response of rat 4 (Table 1; Figure 1 B, C). Thus, if the present contrasting findings might be repeated for ochratoxin A, assumptions about the validity of extrapolating from rat renal carcinogenicity data to risk assessment concerning this mycotoxin for humans may be questionable.
